# Importation, circulation, and emergence of variants of SARS-CoV-2 in the South Indian state of Karnataka

**DOI:** 10.12688/wellcomeopenres.16768.2

**Published:** 2022-02-07

**Authors:** Chitra Pattabiraman, Pramada Prasad, Anson K. George, Darshan Sreenivas, Risha Rasheed, Nakka Vijay Kiran Reddy, Anita Desai, Ravi Vasanthapuram

**Affiliations:** 1 Neurovirology, National Institute of Mental Health and Neurosciences, India, Bangalore, Karnataka, 560029, India; 2 Nodal Officer Genetic Confirmation of SARS-CoV-2, Government of Karnataka, Bengaluru, India

**Keywords:** SARS-CoV-2, variants, Variants of Concern, VOC, COVID-19, COVID-19 India, Karnataka, India, genomic epidemiology, outbreak investigation, SARS-CoV-2 spread, SARS-CoV-2 variants of concern

## Abstract

**Background:** As the coronavirus disease 2019 (COVID-19) pandemic continues, the selection of genomic variants of severe acute respiratory syndrome coronavirus 2 (SARS-CoV-2) associated with higher transmission, more severe disease, re-infection, and immune escape are a cause for concern. Such variants have been reported from the UK (B.1.1.7), South Africa (B.1.351) and, Brazil (P.1/B.1.1.28). We performed this study to track the importation, spread, and emergence of variants locally.

**Methods:** We sequenced whole genomes of SARS-CoV-2 from international travellers (n=75) entering Karnataka, South India, between Dec 22, 2020 and Jan 31, 2021, and from positive cases in the city of Bengaluru (n=108), between Nov 22, 2020- Jan 22, 2021, as well as a local outbreak. We present the lineage distribution and analysis of these sequences.

**Results:** Genomes from the study group into 34 lineages. Variant B.1.1.7 was introduced by international travel (24/73, 32.9%). Lineage B.1.36 and B.1 formed a major fraction of both imported (B.1.36: 20/73, 27.4%; B.1: 14/73, 19.2%), and circulating viruses (B.1.36: 45/103; 43.7%,. B.1: 26/103; 25.2%). The lineage B.1.36 was also associated with a local outbreak. We detected nine amino acid changes, previously associated with immune escape, spread across multiple lineages. The N440K change was detected in 45/162 (27.7%) of the sequences, 37 of these were in the B.1.36 lineage (37/65, 56.92%)

**Conclusions:** Our data support the idea that variants of concern spread by travel. Viruses with amino acid replacements associated with immune escape are already circulating. It is critical to check transmission and monitor changes in SARS-CoV-2 locally.

## Introduction

The coronavirus disease 2019 (COVID-19) pandemic caused by severe acute respiratory syndrome coronavirus 2 (SARS-CoV-2) has claimed millions of lives and has affected people living in all parts of the globe ^1^. The evolution of the virus did not initially alarm public health specialists or those involved in vaccine development ^2^. However, the emergence of variants with distinct biological properties which include one or more mutations that confer higher infectivity, increased transmission, severe disease, re-infection, and immune escape are a cause for concern ^3– 9^. Such variants may influence the trend of the pandemic and are therefore broadly known as Variants of Concern (VOCs) ^3– 8^.

In India, the COVID-19 pandemic began with the importation of the virus in January 2020 ^10^⁠. It is only after 11 million cases and over 150,000 deaths that the numbers declined, signalling the end of the first wave of SARS-CoV-2 in the country ^1, 10, 11^. As with other countries in the world, India too started vaccination campaigns in January 2021, at about the same time that reports of VOCs were communicated from the United Kingdom (UK), Brazil, and South Africa ^3, 4, 6, 11^. The primary concern is that they may herald the second wave of SARS-CoV-2 in the county and/or undermine the vaccination drive.

Genomic studies in India have shown that several lineages of SARS-CoV-2 have been introduced, have spread, and fallen below the limit of detection since January 2020 ^12– 22^. We have previously performed detailed genomic epidemiology of SARS-CoV-2 in the South Indian state of Karnataka, with a population of 64.1 million (Census 2014) ^22^. We found multiple introductions of SARS-CoV-2 into the state and at least seven distinct lineages were already circulating in the state by May 2020. Detailed analysis of the contact network of COVID-19 cases to look at transmission within the state emphasized the role of symptomatic individuals in spreading the virus ^23^. These data have contributed to our understanding of how the virus enters, spreads, and evolves in a population. In the genomic epidemiology study, no particular lineages were associated with disease severity ^22^. Studies of sequences from India juxtaposed with sequences from all over the world, suggest that mutations associated with immune escape and re-infection are already circulating in the population ^2, 24– 26^.

Multiple lineages of SARS-CoV-2 have been reported from across the world and in India ^12, 13, 15– 17, 19– 22, 27^. There are two ancestral lineages of SARS-CoV-2 in the PANGO classification system, A and B ^28^. While viruses of both lineages are circulating across the world, viruses of lineage B are more widespread and prominent in number. The viruses responsible for the outbreak in Italy, in early 2020, with an amino acid change in the spike protein D614G were classified into lineage B.1 ^28^. This lineage is now the dominant lineage across the world. Several studies have now shown that viruses in this lineage transmit better, with increased infectivity in cell culture ^29– 32^.

Viruses of the lineage B.1 have acquired several other amino acid replacements in the Receptor Binding Domain of the Spike protein – specifically in the lineages which have been designated as VOCs, namely -B.1.1.7 (N501Y), B.1.351 (N501Y, E484K, K417T) and P.1 from the lineage B.1.1.28 (N501Y, E484K, K417T). Some of these amino acid replacements either singly or in combination have been shown to influence transmission of the virus, interfere with neutralization of the virus, and are associated with an increase in the number of hospitalizations ^2, 5, 7, 8^. The spread of these lineages, therefore, has global implications ^5, 33^. Early data suggests that some variants may escape neutralization by both therapeutic antibodies and antibodies induced by previous infection and vaccination ^8, 9, 34, 35^. This has implications for the efficacy of Spike sequence-based vaccines and suggests that re-infection is possible ^7, 36^.

Rapid sharing of genomic information enabled the global community to pick-up cases of VOCs and implement relevant public health measures ^3, 4, 6^. A concentrated, ongoing, local approach to genomic surveillance is critical for the identification of variants and establishing epidemiological links with the trend of the outbreak ^5, 7, 12, 22^. This has also proved critical for local outbreak management and informed policy decisions across the world ^5, 7, 37, 38^.

It is in this context that we conducted genomic surveillance of COVID-19 positive international travellers to the south Indian state of Karnataka between Dec 22, 2020- Jan 31, 2021 (n=75). We also performed sequencing of SARS-CoV-2 (n=108), in samples collected between Nov 22, 2020- Jan 22, 2021) in Bengaluru city (Bengaluru Urban District) to identify and track locally circulating variants and potential VOCs.

## Methods

### Study setting and ethical considerations

The Department of Neurovirology, at the National Institute of Mental Health and Neurosciences (NIMHANS), Bengaluru, is an ICMR (Indian Council of Medical Research) approved COVID-19 diagnostic centre. The Government of Karnataka and the Government of India designated our lab as a nodal centre for genomic sequencing. This study was granted a waiver by the Institutional Ethics Committee of NIMHANS in light of the public health emergency. All samples were collected for routine diagnosis for COVID-19, as part of the State’s requirement for epidemiological investigation of variants; and de-identified before analysis of data.

### Samples for sequencing

On December 23, 2020, the Government of India established surveillance for detecting the importation of Variants of Concern. As part of this surveillance, nasopharyngeal and oro-pharyngeal swabs were collected from international travellers arriving at the international airport in Bengaluru between Dec 22, 2020- Jan 31, 2021. Samples testing positive by reverse transcription polymerase chain reaction (RT-PCR) and having Ct value < 30 (n=75) were included in the study. Further, the surveillance was also designed to include at least 5% of RT PCR positive samples received for routine diagnosis of COVID-19 from Nov 2020 -Jan 2021. To fulfil this samples from COVID-19 cases in Bengaluru city (n=108, 16.25% (108/664) collected between Nov 22, 2020- Jan 22, 2021) through routine surveillance and from a local outbreak in a nursing college in Bengaluru city in Feb 2021, n=14 were included in the study. Of the 42 samples collected from the local outbreak, 14 were suitable for sequencing (RT-PCR positive, Ct value < 30) and were analysed further. From previous experiments in our laboratory using a similar sequencing approach, we have ascertained that a Ct value of < 30 can inform on lineage of the virus and a Ct of < 25 was correlated with recovery of complete genomes. This was used to set the cut-offs for the two sample types.

### Data for epidemiological curve

The data for confirmed new cases and numbers tested for SARS-CoV-2 in Bengaluru Urban district during the study period (Nov 22, 2020 – Jan 31, 2021) were obtained from covid19india.org, a dashboard that collated information released by the Government of Karnataka.

### Nucleic Acid extraction and RT-PCR

Nucleic acid extraction was performed with automated magnetic bead-based extraction method, using the Chemagic Viral DNA/RNA special H96 kit (PerkinElmer, CMG-1033-S) following manufacturer’s instruction. SARS-CoV-2 detection was done using ICMR approved diagnostic kits. A total of 197 RT-PCR positive samples fulfilling the following criteria – (i) Ct values less than 30 in the case of international travellers (n=75), and local outbreak (n=14) or (ii) Ct value less than 25 for local cases (n=108), were taken for whole genome sequencing. Samples and RNA were stored at 4C for <1 week and -80C for long term storage.

### Whole genome sequencing of SARS-CoV-2

Whole genome sequencing was performed using the amplicon sequencing approach described in the ARTIC Network protocol using the V3 primer set ^39^. The resulting amplicons from 12–24 samples were barcoded using the native barcoding kits (NBD104/114, Oxford Nanopore Technology (ONT)) and sequencing libraries were prepared using the ligation sequencing kit (SQK-LSK109, ONT). The barcoded library was loaded on to FLO-MIN-106 flow cells and sequenced on the MinION (ONT). An average of 0.12 million (median) sequencing reads were acquired per sample with a median coverage of 1737x (see extended data, S1 ^40^). Raw sequencing reads have been deposited within BioProject ID: PRJNA670824.

### Analysis of sequencing data and data sharing

Analysis of sequencing reads was performed as described previously ^22^. Briefly, sequences were basecalled and demultiplexed using guppy (v3.6) from ONT, alternatively open source basecallers such as Bonito can be used Sequences of length 100–600bp were considered for further analysis. Primer sequences were removed from the sequencing reads by trimming 25bp at the ends and additional trimming based on alignment using BBDuk (v38.37). Resulting reads were mapped to SARS-CoV-2 reference genome (NC_045512) using Minimap2 (v2.17) within Geneious Prime (Geneious Prime 2020.0.3). A consensus was created by calling the majority base (most common base) at each position, resulting in a consensus with the lowest ambiguities. Regions with coverage lower than 10 reads were called Ns. The consensus was aligned to the reference to ensure the correct reading frame, edited by manual inspection of the contigs, followed by transfer of annotations from the reference sequence. Of the 183 samples from international travellers and local cases, 176 (73/75 imported, 103/108 circulating) genomes could be used for the determination of lineage using the PANGO web application (Pangolin v2.2.2 lineages version 2021-02-12) ^28^. Of the 176 genomes,162 were complete (>92% at 1X and >85% at 10X) and were deposited into the GISAID Database ^41^, accession numbers for the sequences are provided as extended data (see extended data S2 ^40^). Sequences have also been deposited in GenBank with accession numbers OM073810 – OM073973. Complete sequences (162) were analysed for SNPs and amino acid replacements with reference MN908947.3 (Wuhan-Hu-1) using the CoV-Glue Web Application ^42^.

### Phylogenetic analysis

A total of 168 genomes, including the 162 described above, and an additional 6 complete genomes from a local outbreak, were used for phylogenetic analysis with the reference NC_045512 as an outgroup. Multiple sequence alignment was performed using MUSCLE and a maximum likelihood tree was constructed using iqtree ^43, 44^. The GTR+F+I+G4 substitution model was found to be the best-fit model (of the 88 models tested) using the Bayesian Information Criterion. The consensus tree was constructed from 1000 bootstraps and bootstrap values over 70 were interpreted.

## Results

**Figure 1.**
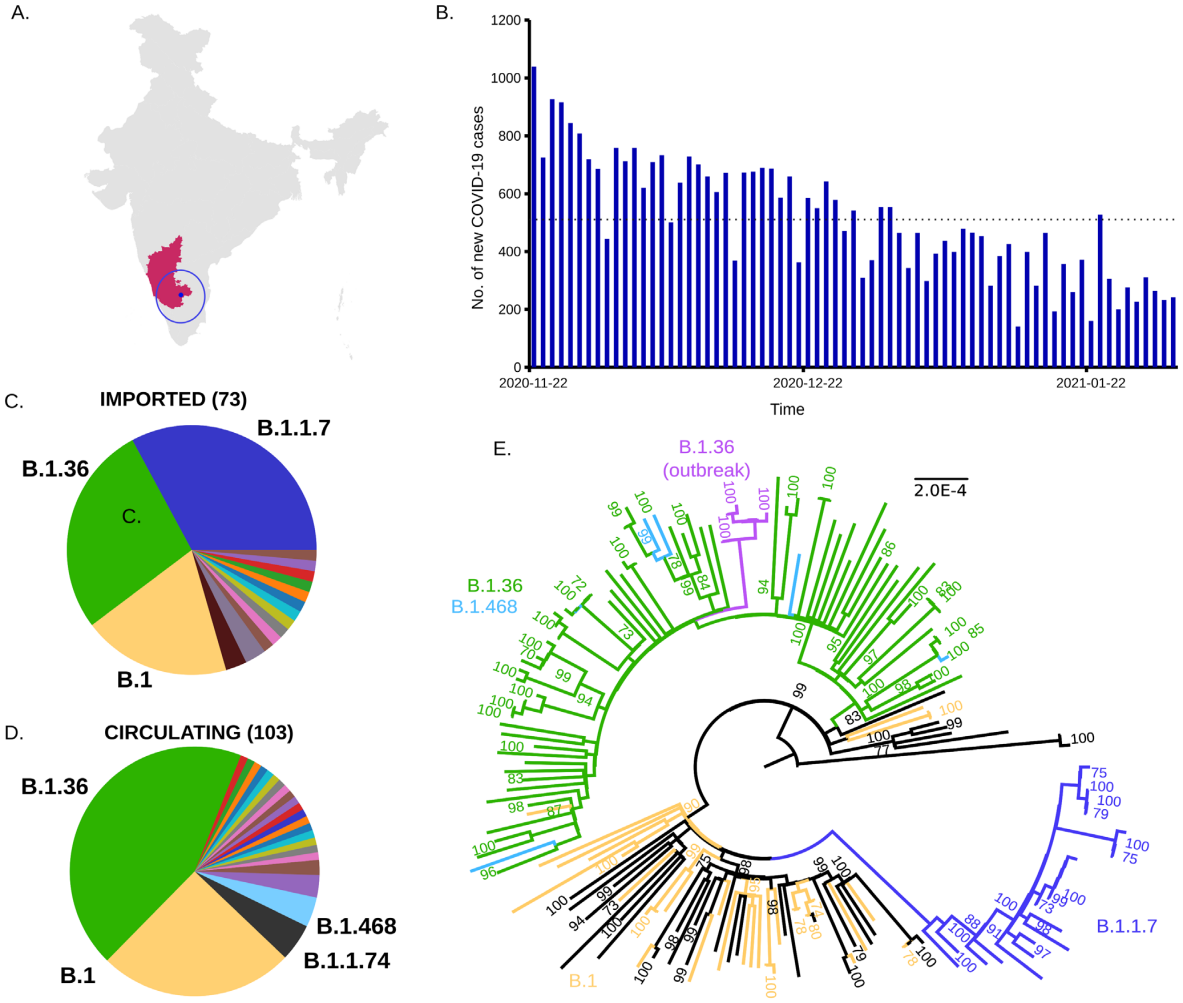
Distribution of SARS-CoV-2 Lineages in the state of Karnataka (Nov 22, 2020- Jan 31, 2021). **A**) Geographical location of Bengaluru city (blue dot, highlighted by blue circle) in the South Indian state of Karnataka (in pink) is shown on the map of India. ( **B**) Epidemiological curve of COVID-19 cases in Bengaluru Urban district in the study period. Dotted horizontal line is the average daily cases for the time period. ( **C**) Severe acute respiratory syndrome coronavirus 2 (SARS-CoV-2) lineages imported by international travel into Karnataka (n= 73). ( **D**) SARS-CoV-2 lineages circulating in Bengaluru city (n= 103). Colours represent different lineages. Lineages with greater > 4 sequences are labelled. ( **E**) Maximum Likelihood Phylogenetic tree of 168 complete SARS-CoV-2-genomes from Karnataka, rooted by the reference genome (NC_045512). The scale (length of branches) is in substitutions per nucleotide site. Predominant lineages are coloured. Sequences from a local outbreak of SARS-CoV-2 are coloured in magenta. Numbers on the nodes indicate bootstrap support values (in %), values above 70 are shown.

In the study period (Nov 22, 2020 – Jan 21, 2021) an average of 510 SARS-CoV-2 cases were detected daily in the district of Bengaluru Urban in the South Indian state of Karnataka ( Figure 1A, B). We sequenced SARS-CoV-2 genomes from 197 SARS-CoV-2 positive individuals, including international travellers (n=75), local cases (n=108), and a local outbreak (n=14). Lineage classification using the PANGO scheme was possible for 176 genomes which were either imported (73/75) or circulating (103/108), and for all 14 genomes from the local outbreak (extended data S3 ^40^). The genomic surveillance for the local outbreak was carried out to identify the lineage/lineages responsible for the outbreak ( Figure 1CE).

A total of 34 lineages were detected from the 176 genomes in this study. A complete list of lineages and their frequencies are tabulated in Table 1. Briefly, genomes from imported and circulating viruses belong to both A (3/176) and B (173/176) lineages. Within A, two (2/103) circulating genomes were classified into A.23.1. Of the 173 genomes in lineage B, two genomes were classified into lineage B (2/173), the rest were derived from B.1 (130/173) or B.1.1 (41/173).

**Table 1.** PANGO lineage assignments for SARS-CoV-2 genomes. Frequency of SARS-CoV-2 lineages (PANGO classification) and percentages of sequenced samples in the imported (by international travel, n =73) or already circulating (in Bengaluru city, n=103) are tabulated. Lineages in black are present in both categories, blue were found in the imported group and orange in circulating viruses. Sub-lineages of B.1.36 are highlighted.

	Lineage	IMPORTED		CIRCULATING	
	(PANGO)	Dec 22, 2020 – Jan 31, 2021		Nov 22, 2020 – Jan 22, 2021	
		Frequency	Percentage	Frequency	Percentage
1	B.1.1.7	24	32.9	1	1.0
2	B.1.36	20	27.4	45	43.7
3	B.1	14	19.2	26	25.2
4	B.1.177	2	2.7		
5	B.1.468	2	2.7	4	3.9
6	A	1	1.4		
7	B	1	1.4	1	1.0
8	B.1.1.194	1	1.4		
9	B.1.1.317	1	1.4		
10	B.1.1.74	1	1.4	5	4.9
11	B.1.177.19	1	1.4		
12	B.1.177.4	1	1.4		
13	B.1.2	1	1.4		
14	B.1.258	1	1.4		
15	B.1.308	1	1.4		
16	B.1.36.18	1	1.4		
17	A.23.1			2	1.9
18	B.1.1.106			1	1.0
19	B.1.1.130			1	1.0
20	B.1.1.184			1	1.0
21	B.1.1.197			1	1.0
22	B.1.1.216			3	2.9
23	B.1.1.306			1	1.0
24	B.1.197			1	1.0
25	B.1.216			1	1.0
26	B.1.221			1	1.0
27	B.1.256			1	1.0
28	B.1.36.10			1	1.0
29	B.1.36.13			1	1.0
30	B.1.36.17			1	1.0
31	B.1.36.23			1	1.0
32	B.1.456			1	1.0
33	B.1.509			1	1.0
34	B.1.188			1	1.0

The genomes from imported cases grouped into 16 distinct lineages ( Figure 1A, Table 1) including B.1.1.7 (24/73, 32.9%), B.1.36 (20/73, 27.4%) and B.1 (14/73, 19.2%). The first introduction of B.1.1.7 was noted in the last week of December 2020, and by January 31, 2021, this lineage made up 32.9% (24/73) of all imported cases ( Figure 1AC, Table 1). Circulating genomes grouped into 24 distinct lineages, dominated by the lineages B.1.36 (45/103; 43.7%), B.1 (26/103; 25.2%), B.1.1.74 (5/103; 4.9%) and B.1.468 (4/103; 3.9%) ( Figure 1BD, Table 1). Only a single sequence of B.1.1.7 was detected during the study period as part of this surveillance effort in a non-traveller. Sequences from the lineage B.1.36 and derived lineages (70/176) grouped into a distinct phylogenetic clade together with sequences belonging to lineage B.1.468 (6/176) ( Figure 1CE). Phylogenetic analysis of a sub-set of B.1.36 genomes from across India and across the world showed that most of the sequences both from people with a history of international travel as well as circulating viruses clustered together (extended data SF1 ^40^).

Genomic investigation of an outbreak of SARS-CoV-2 in the city of Bengaluru in early Feb 2021, revealed that 14/14 sequences (all 14 genomes had 1X coverage >74%.) from the outbreak could be classified into lineage B.1.36. Complete genome sequences (with >92% coverage of reference at 1X and >85% at 10X) could be recovered from 6/14 cases. All six viruses grouped into a clade within the largely B.1.36+B.1.468 clade ( Figure 1CE).

Of the 176 genomes from travellers and in circulation, for which lineage classification was possible,162 complete genomes (with coverage > 92% at 1X and > 85% at 10X) were used for the analysis of SNPs and amino acid replacements. A total of 968 SNPs (extended data, S4 ^40^) and 529 amino acid replacements (extended data S5 ^40^) were identified. Of these amino acid replacements 61 were in the Spike protein of circulating viruses, and 32 in Spike protein of imported viruses (extended data S6 ^40^). The B.1.36 lineage had 226 amino acid replacements, 31 of these were in the Spike protein. Although only the D614G and N440K were present in an appreciable number (>50%) of sequences (extended data S7 ^40^. The N440K was found in 37/65 (56.92%) of B.1.36 sequences (extended data S7 ^40^).

**Figure 2.**
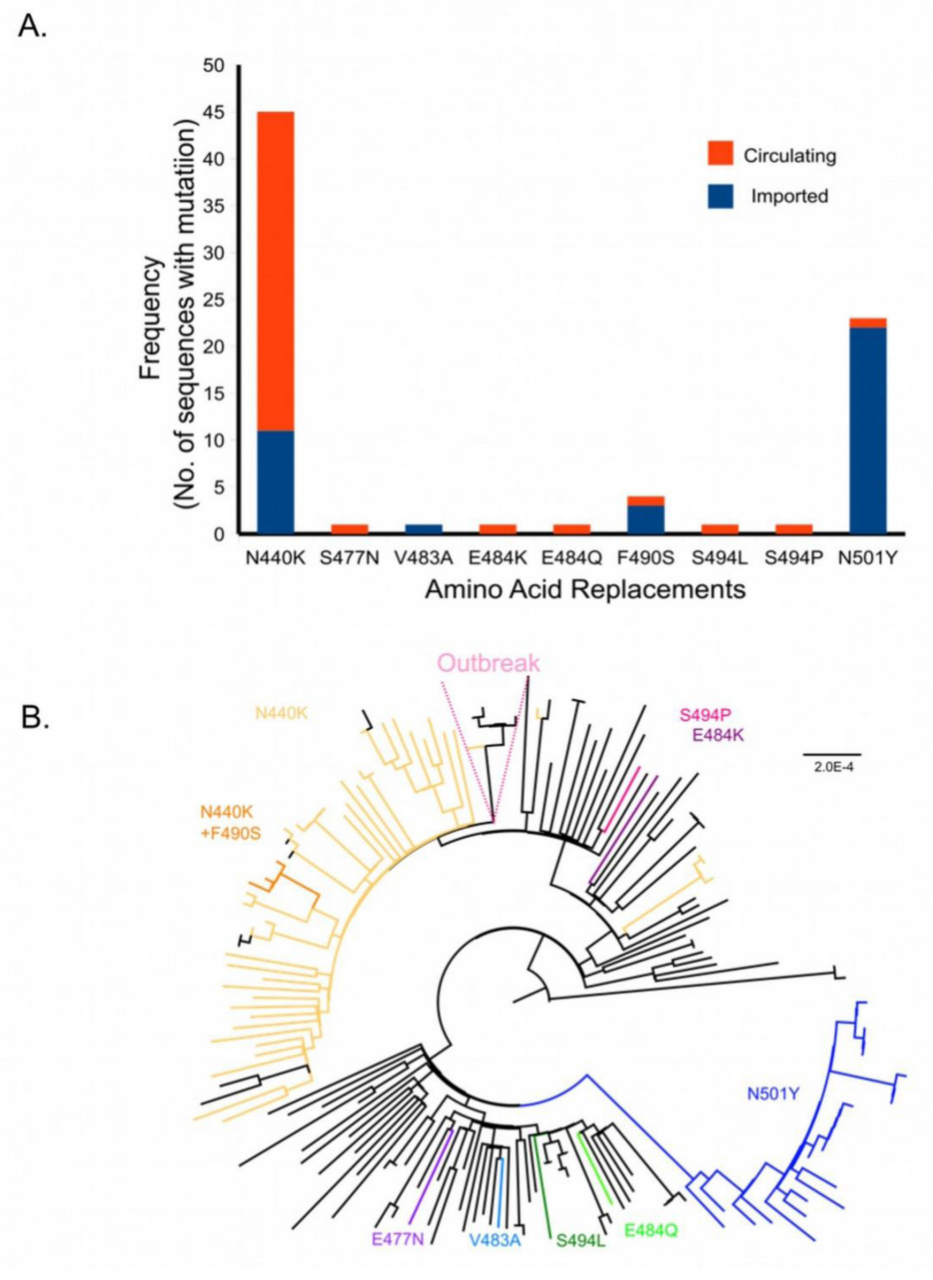
Amino acid replacements in the Receptor Binding Domain (RBD) of severe acute respiratory syndrome coronavirus 2 (SARS-CoV-2). ( **A**) Frequency of amino acid replacements in the RBD (amino acids- 387- 516 in the Spike protein) is shown as a bar graph. Frequencies are plotted against the amino acid replacement. Orange and blue represent the circulating and imported genomes respectively. ( **B**) Maximum Likelihood phylogenetic tree highlighting branches with the indicated amino acid substitutions.

We carried out further analysis of the amino acid replacements in the receptor binding domain (RBD) of the spike protein ( Figure 2A, extended data, S6 ^40^) and mapped them on the Maximum-Likelihood tree ( Figure 2B). We identified mutations leading to nine amino acid replacements in the RBD ( Figure 2A). Of these, five (S477N, E484K, E484Q, S494L, S494P) were found in viruses circulating in Bengaluru, and the amino acid replacement V483A was from an imported case. The N501Y change was confined to the B.1.1.7 lineage. The N440K mutation was present in 45/162 (27.7%) of the sequences. All 45 sequences with N440K are grouped into the B.1.36+B.1.468 clade ( Figure 2B). Of the six sequences from a cluster of cases (Outbreak), only a single sequence carried the mutation resulting in the N440K change ( Figure 2B). A single branch of the B.1.36+B.1.468 clade (n=4, 3 of which were imported) had an additional amino acid replacement F490S in the RBD ( Figure 2B). The mutations in the RBD were seen across the phylogenetic tree and clades ( Figure 2B).

## Discussion

In this study, we found 34 lineages of SARS-CoV-2 circulating and imported into Bengaluru city in Karnataka, India, between Nov 22, 2020 – Jan 31, 2021. We aimed to detect the introduction of the global VOCs (lineages B.1.1.7, B.1.351, P.1/B.1.1.28), as well as genotype the variants of SARS-CoV-2, circulating since our last study, which highlighted the introduction and spread of seven lineages of SARS-CoV-2 in Karnataka, between March-May 2020 ^22^.

We found no evidence suggesting that the B.1.1.7 lineage was present in Karnataka before late-Dec 2020. We first detected the B.1.1.7 variant in Karnataka, in an international traveller from a sample collected on Dec 22, 2020 (extended data, S3 ^40^). The first and only case of non-travel related B.1.1.7, in our study, was detected in the middle of Jan 2021 in an individual who was in contact with an international traveller (extended data, S3 ^40^). These data together suggest that B.1.1.7 in Karnataka was limited to travel-associated cases and was not in the community during the study period. At the end of the study period, the B.1.1.7 lineage was detected in 32.9% of all imported cases ( Table 1). We did not detect the variants P.1/B.1.1.28 or B.1.351 reported from Brazil and South Africa respectively in this study.

We found that B.1.36 and B.1 lineages dominated in both the imported (20/73; 27.4%,14/73, 19.2%) and circulating viruses (45/103; 43.7%, 26/103; 25.2%) in our study ( Table 1). The clustering of B.1.36 lineage from travellers with locally circulating viruses suggests either a common source of infection or infection with circulating lineages post-arrival (extended data SF1). B.1.36 was first reported from Saudi Arabia in Feb 2020 (extended data S8 ^40^ ( Table 1). The B.1.36 lineage was both imported by international travel (20/73) and circulating (45/103) in Bengaluru city ( Table 1). The lineage is characterized by the following amino acid replacements- nsp12-P323L(95.38%), S-D614G (93.85%), S-N440K (56.92%), ORF 3a-Q57H (90.77%), ORF 3a-E261*(81.54%), nsp3-T183I (81.54%), nsp16-L126F(80%), N-S2P (72.31%), ORF 8-S97I (72.31%) (extended data, Supplementary Table 7 ^40^). The immune escape associated amino acid change, N440K has been reported from the states of Andhra Pradesh, Maharashtra, Telangana, and Karnataka, and is also associated with reinfection ^24, 36, 45^. This change was found in 37/65 (56.92%) of the sequences clustering to B.1.36 (extended data S7 ^40^). Sequencing from Bengaluru Urban district, which encompasses Bengaluru city, during this time period is sparse. Analysis of a limited number of sequences (n= 649 between 23 Nov 2020 - May 2 2021) from our laboratory suggest that B.1.36 and its sub lineages dominated in late 2020, co-circulated with B.1.1.7/Alpha and B.1.617.1/Kappa between Jan-Mar 2021 and were displaced by B.1.617.2/Delta by April-May 2021 (extended data SF2).

An outbreak of SARS-CoV-2 occurred in Bengaluru in early Feb 2021, raising concerns about the spread of variants, the threat of a second wave, and reduction in the efficacy of vaccines. This outbreak in a college where students were returning from different states within India was driven by related viruses belonging to the B.1.36 lineage ( Figure 1CE and extended data, S3 ^40^). Only one of the six sequences from the outbreak cluster had the mutation resulting the N440K replacement in the Spike protein ( Figure 2C, extended data S6

Apart from the introduction and spread of known VOCs, the emergence of variants locally is also a cause for concern. Early in the pandemic, a single mutation in the gene encoding the Spike protein of SARS-CoV-2 resulting in a D614G amino acid change was identified to increase infectivity and transmission ^2, 29, 32^. Viruses with this amino acid replacement dominate across the globe ^31, 46^. Mutations in the gene encoding the Spike protein are of particular concern due to the role of this protein and its Receptor Binding Domain (RBD) in viral binding and entry ^47^. Some of these mutations have been shown to increase infectivity, affinity to the angiotensin converting enzyme 2 (ACE-2) receptor or affect neutralization by antibodies i *n vitro.* Viral genomes with these mutations were already circulating viruses by mid-2020 ^2, 25, 26, 45, 48, 49^.

In the sequences from this study, nine amino acids replacements were noted in the RBD domain of the Spike protein ( Figure 2B and extended data S6 ^40^). They occurred singly or in pairs (N440K+F490S) ( Figure 2). All nine amino acid changes, namely N440K, S477N, V483A, E484K/Q, F490S, S494L/P, N501Y are associated with immune escape ^24, 25^. Viruses with some of these amino acid changes were already known to be circulating in other parts of India ^16, 17, 24^.

Mutations in the gene encoding Spike protein that do not map to the RBD have also been described; particularly near the polybasic cleavage site at the S1/S2 boundary of the Spike protein. Towards the end of the year 2020, multiple lineages with amino acid replacements at position 677 were noted ^50^. Four viruses in our study have mutations resulting in amino acid changes at this position (Q677H (n=3), Q677P (n=1)) (extended data, S6 ^40^).

It is to be noted that in this study we have only included samples with Ct values less than 25 for surveillance of circulating SARS-CoV-2 genomes and Ct values less than 30 for sequencing of international travel-related cases. We have also sequenced only a fraction of cases in a limited geographical area. This may therefore present an incomplete view of circulating viruses and inflate the ones that are more readily sequenced. Also, as we have used the amplicon sequencing approach, not all regions of all lineages are well covered by sequencing reads. Others have also noted homoplasy in SARS-CoV-2, this highlights the need to be cautious while interpreting the phylogenetic relationships between SARS-CoV-2 sequences, especially in the context of outbreaks ^51^.

In summary, our data highlight an increase in the frequency of the lineage B.1.36 in Bengaluru Urban, in Karnataka, and indicate an underappreciated global presence of this lineage ( Figure 1, Table 1, extended data S8 ^40^). Whether this increase is because of epidemiological linkages such as increased travel, continued local transmission chains or super-spreader events remains to be determined. It is beyond the scope of this work to examine whether the lineage, contributing mutations, and amino acid changes impact transmission/infectivity of the virus. Our data emphasize that a consolidated and local approach to genomic surveillance which includes sequencing of SARS-CoV-2 from travellers, circulating variants, and outbreaks, in a continuous manner is necessary to detect VOCs. We believe that in regions where sequencing capacity is limited and sustained and continuous sequencing of local cases is a challenge, rapid and focussed sequencing of travellers and local outbreaks may serve as early warning systems for novel variants. Even as this conjecture remains to be tested, it is clear that rapid identification of such variants can aid in preparing the healthcare system for a surge in cases, suggest revisions to vaccines and diagnostic tests, inform the international community, and guide public health measures.

## Data availability

### Underlying data

NCBI BioProject: SARS-CoV-2 Genome Sequencing. Accession number PRJNA670824; https://identifiers.org/NCBI/bioproject:PRJNA670824.

### Extended data

Open Science Framework: SARS-CoV-2 Sequencing.

DOI https://doi.org/10.17605/OSF.IO/S56BR ^40^.

This project contains the following extended data:

S1: Summary of sequencing results

S2: GISAID Accession ID for sequences

S3: Lineage, source and collection date of sequenced samples

S4: Position and frequency of single nucleotide polymorphisms

S5: Position and frequency of amino acid replacements

S6: Amino acid replacement in Spike protein

S7: Frequency of amino acid replacements in lineage B.1.36

S8: Location and Sequence counts for B.1.36 between Nov 22, 2020 – Jan 31, 2021

S9: B.1.36 Sequence GISAID Submitters_acknowledgement

SF1: Phylogenetic tree of a subset of B.1.36 sequences (Nov 2020 - Jan 2021)

SF2: Distribution of SARS-CoV-2 lineages in Bengaluru Urban (Nov 23, 2020 – May 2, 2021)

Data are available under the terms of the Creative Commons Attribution 4.0 International license (CC-BY 4.0).
