# Supplementary material for: Importation, circulation, and emergence of variants of SARS-CoV-2 in the South Indian state of Karnataka
Source: Wellcome Open Res. 2022 Feb 7;6:110. Originally published 2021 May 13. [Version 2] doi: 10.12688/wellcomeopenres.16768.2 (PMC8857524; doi:10.12688/wellcomeopenres.16768.2)
Supplement: Peer review history

# Peer review: Reviewer response for version 2

**DOI:** 10.21956/wellcomeopenres.19557.r48535

**Published:** 2022-02-18

**Authors:** Richard Orton

**Affiliations:** 1 MRC-University of Glasgow Centre for Virus Research, Glasgow, UK

The authors have addressed all my previous comments. Two minor points:

1. References should be used for the tools used such as minimap2, etc. - there are papers for these tools.
2. The bioinformatics section should have the "manual inspection/curation" line expanded a little bit further to state that you specifically checking indwells and manually inspecting for underlying read support.

Is the work clearly and accurately presented and does it cite the current literature?

Yes

If applicable, is the statistical analysis and its interpretation appropriate?

Not applicable

Are all the source data underlying the results available to ensure full reproducibility?

Yes

Is the study design appropriate and is the work technically sound?

Yes

Are the conclusions drawn adequately supported by the results?

Yes

Are sufficient details of methods and analysis provided to allow replication by others?

Yes

Reviewer Expertise:

viral bioinformatics, inter and intra-host viral evolution

I confirm that I have read this submission and believe that I have an appropriate level of expertise to confirm that it is of an acceptable scientific standard.

# Peer review: Reviewer response for version 1

**DOI:** 10.21956/wellcomeopenres.18493.r46594

**Published:** 2021-11-09

**Authors:** Richard Orton

**Affiliations:** 1 MRC-University of Glasgow Centre for Virus Research, Glasgow, UK

Overall, I found this to be a very well written and interesting paper. I do have a number of minor points to address:

- Abstract - (B.136: 20/73, 27.4%; B.1: 14/73, 19.2%) - B.1.36 not B.136?
- Introduction - "The viruses responsible for the catastrophic outbreak in Italy," - I have not seen this outbreak in Italy described as "catastrophic" before, and almost suggests this is the cause of the pandemic rather than the initial outbreak in China.
- Methods - Analysis of seq data and data sharing - this point is quite important:
- The ONT sequencing was done using the artic protocol and artic v3 primers. The bioinformatics, however, does not use the recommended artic protocol: https://artic.network/ncov-2019/ncov2019-bioinformatics-sop.html. There needs to be more information on how the bioinformatics was done, specifically this step "A consensus genome was generated with a coverage cut-off of 10x and the 0% majority rule."
- What tool was used to generate this? What does a 0% majority's rule mean?
- To put into context the artic pipeline using a 40x cutoff for consensus calling, and the variant calling goes through nanopolish to address the highly problematic indels from ONT data, there are other steps as well to remove reads of abnormal length, and remove reads aligning to unexpected regions - were any of these steps done? If not are you sure of the reliability of your consensus seqs? How many of your consensus sequences have indels which broke coding sequences?
- Results - "Genomic investigation of an outbreak of SARS-CoV-2 in the city of Bengaluru in early Feb 2021, revealed that 14/14 sequences from the outbreak could be classified into lineage B.1.36. Complete genome sequences could be recovered from 6/14 cases." How can all 14 be classified as B.1.36 if only 6/14 could have a complete genome recovered?
- Overall - Lineage B.1.36 - I found it quite surprising that B.1.36 formed a large proportion of importations. This seems a relatively small lineage (and just checking it out quickly seems only really observed at a decent freq in India, Canada, and Hong Kong - and to a lesser degree the UK) - so it seems quite surprising that this is the dominant lineage imported from international travellers? I think it would be very useful to have a breakdown of the country of origin the travellers have come from - is this possible? How many of the travellers are tourists and how many are returning travellers (would they have been tested before travelling out) And/Or - A table of B.1.36 counts from countries around the world for the weeks preceding and during the study.
- Context - some extra elements for general context might be useful - a map showing the geographical location of the region, an epidemic curve showing the number of COVID-19 cases (& genomes sequenced) in India and the region in question, and the dominant lineages over time - in particular, B.1.36 but also B.1.1.7 and later delta - did B.1.36 persist after the introduction of B.1.1.7 and then the subsequent emergence of delta, etc
- As the mutation N440K is a main point of the paper - how frequently is this mutation observed across lineages and within B.136 (all seqs)? The N440K mutation does not seem be a lineage defining mutation of B.1.36 - but the majority of your seqs seem to have it.

Is the work clearly and accurately presented and does it cite the current literature?

Yes

If applicable, is the statistical analysis and its interpretation appropriate?

Not applicable

Are all the source data underlying the results available to ensure full reproducibility?

Yes

Is the study design appropriate and is the work technically sound?

Yes

Are the conclusions drawn adequately supported by the results?

Yes

Are sufficient details of methods and analysis provided to allow replication by others?

Yes

Reviewer Expertise:

viral bioinformatics, inter and intra-host viral evolution

I confirm that I have read this submission and believe that I have an appropriate level of expertise to confirm that it is of an acceptable scientific standard, however I have significant reservations, as outlined above.

# Response

**Published:** 2022-01-30

**Authors:** Chitra Pattabiraman

We thank the reviewer for accepting and taking the time to review this manuscript during the pandemic and for their considered comments. We have made changes to the manuscript based on their recommendations. Please find a point-wise response to the comments below with changes to the manuscript indicated

**1.Abstract - (B.136: 20/73, 27.4%; B.1: 14/73, 19.2%) - B.1.36 not B.136?**

 This has been corrected.

**2. Introduction - "The viruses responsible for the catastrophic outbreak in Italy," - I have not seen this outbreak in Italy described as "catastrophic" before, and almost suggests this is the cause of the pandemic rather than the initial outbreak in China.**

 The word catastrophic has been removed.

**3. Methods - Analysis of seq data and data sharing - this point is quite important:**

**The ONT sequencing was done using the artic protocol and artic v3 primers. The bioinformatics, however, does not use the recommended artic protocol:** **https://artic.network/ncov-2019/ncov2019-bioinformatics-sop.html** **. There needs to be more information on how the bioinformatics was done, specifically this step "A consensus genome was generated with a coverage cut-off of 10x and the 0% majority rule."**

 The methods section has been changed to include a detailed bioinformatic workflow.

 “Briefly, sequences were basecalled and demultiplexed using guppy (v3.6) from ONT, alternatively open source basecallers such as Bonito can be used Sequences of length 100–600bp were considered for further analysis. Primer sequences were removed from the sequencing reads by trimming 25bp and additional trimming based on alignment using BBDuk (v38.37). Resulting reads were mapped to SARS-CoV-2 reference genome (NC_045512) using Minimap2 (v2.17) within Geneious Prime (Geneious Prime 2020.0.3). A consensus was created by calling the majority base (most common base) at each position, resulting in a consensus with the lowest ambiguities. Regions with coverage lower than 10 reads were called Ns. The consensus was aligned to the reference to ensure the correct reading frame, edited by manual inspection of the contigs, followed by transfer of annotations from the reference sequence.”

**4. What tool was used to generate this? What does a 0% majority's rule mean?**

 A mapping based assembly was performed using minimap2 to the SARS-CoV-2 reference genome (NC_045512). The consensus generation was performed using Geneious Prime® 2020.0.3. The 0% majority rule within Geneious Prime is the following - the most common base at position is called, resulting in a consensus with the lowest ambiguities. A coverage cut-off of 10X was set in order to call the consensus. Regions with coverage lower than 10 were called Ns.

 We have changed this sentence in the Methods (described above) for clarity.

**5. To put into context the artic pipeline using a 40x cutoff for consensus calling, and the variant calling goes through nanopolish to address the highly problematic indels from ONT data, there are other steps as well to remove reads of abnormal length, and remove reads aligning to unexpected regions - were any of these steps done? If not are you sure of the reliability of your consensus seqs? How many of your consensus sequences have indels which broke coding sequences?**

 We agree with the reviewer that these are important considerations to generate a good quality consensus. As described above in the changes to the methods section - adapter and barcode removal, primer trimming and length filters were used in the analysis workflow.

 Due to the computational resources needed for nanopolish, we chose to manually inspect the contigs for indels. The following rules were followed during manual inspection-

 The indels that were due to sequencing errors tended to be poorly supported/regions of heterogeneity and caused frameshifts. We manually inspected the contig for frameshifts. If the frameshift was due to an indel, we inspected the supporting reads. If >70% of the reads had the indel, the consensus was left unchanged. If the indel was not well supported and there were at least 10 reads without the indel, then the majority base was called. In case of a tie, the consensus was resolved to the wild-type/reference base. If less than 10 reads were present, the consensus was corrected to an N. If the indel was present at the end of reads, only the bridging reads were considered for calling the consensus using the above rules.

 We have previously validated this approach using a few samples with nanopolish and are therefore confident of the consensus calls. We also noted that the indels occurred at specific positions in the genome, depending on the genomic context - usually homopolymer repeats or end of reads. These positions that needed manual editing were conserved for a particular lineage and were roughly about 29-30 positions per assembly. Also, both the phylogenetics and Nextclade QC did not suggest major issues with our consensus sequences. Additionally we also provide the sequencing reads in the SRA database for others to rebuild the consensus.

**6. Results - "Genomic investigation of an outbreak of SARS-CoV-2 in the city of Bengaluru in early Feb 2021, revealed that 14/14 sequences from the outbreak could be classified into lineage B.1.36. Complete genome sequences could be recovered from 6/14 cases." How can all 14 be classified as B.1.36 if only 6/14 could have a complete genome recovered?**

 We call genomes complete if consensus covers >85% of reference at 10X and >92% of reference at 1X. Some genomes were not complete by this criteria (extended data S1 and S3), however they had enough information to be assigned a lineage using the Pangolin tool. All 14 genomes had 1X coverage >74%. This now indicated in the text.

**7. Overall - Lineage B.1.36 - I found it quite surprising that B.1.36 formed a large proportion of importations. This seems a relatively small lineage (and just checking it out quickly seems only really observed at a decent freq in India, Canada, and Hong Kong - and to a lesser degree the UK) - so it seems quite surprising that this is the dominant lineage imported from international travellers? I think it would be very useful to have a breakdown of the country of origin the travellers have come from - is this possible? How many of the travellers are tourists and how many are returning travellers (would they have been tested before travelling out) And/Or - A table of B.1.36 counts from countries around the world for the weeks preceding and during the study.**

 We thank the reviewer for the opportunity to discuss this further. We do not have a detailed travel history for the international travellers, however, people with a history of travel to the UK were prioritized for sequencing at that time. The lineage B.1.36 has been detected in 67 countries (with >5% prevalence Saudi Arabia, Iran and Afghanistan) based on GISAID data collated by outbreak.info and has been circulating since February 2020. A cumulative table B.1.36 sequences based on location between Nov 22, 2020 - Jan 21, 2021 is provided in extended data S8. This point is addressed further in response to comment 2 from reviewer 1 and in extended data SF1.

**8. As the mutation N440K is a main point of the paper - how frequently is this mutation observed across lineages and within B.136 (all seqs)? The N440K mutation does not seem be a lineage defining mutation of B.1.36 - but the majority of your seqs seem to have it.**

 The 440K is not a lineage defining mutation for B.1.36. In our study 45/176 genomes had this mutation. The 440K substitution was observed in 37/65 (56.92%) B.1.36 genomes. The following changes have been made to highlight this in the results section-

 “The N440K change was detected in 45/162 (27.7%) of the sequences, 37 of these were in the B.1.36 lineage (37/65, 56.92%).”

 “The N440K was found in 37/65 (56.92%) of B.1.36 sequences (extended data S7).”

 “The N440K mutation was present in 45/162 (27.7%) of the sequences. All 45 sequences with N440K are grouped into the B.1.36+B.1.468 clade (Figure 2B).”

**9. Context - some extra elements for general context might be useful - a map showing the geographical location of the region, an epidemic curve showing the number of COVID-19 cases (& genomes sequenced) in India and the region in question, and the dominant lineages over time - in particular, B.1.36 but also B.1.1.7 and later delta - did B.1.36 persist after the introduction of B.1.1.7 and then the subsequent emergence of delta, etc**

 Fig1 has been modified to include a map of India, showing Karnataka and Bengaluru city. An epi curve for Bengaluru Urban (No. of new cases detected daily during the study period) has also been included as Fig 1B. The sequencing in this region has not been uniform and continuous, however, the data indicate displacement of B.1.36 by Delta by April 2021 (extended data SF2).

 The following line has been added to the text to provide the context

 “Sequencing from Bengaluru Urban district, which encompasses Bengaluru city, during this time period is sparse. Analysis of a limited number of sequences (n= 649 between 23 Nov 2020 - May 2 2021) from our laboratory suggest that B.1.36 and its sub lineages dominated in late 2020, co-circulated with B.1.1.7/Alpha and B.1.617.1/Kappa between Jan-Mar 2021 and were displaced by B.1.617.2/Delta by 'April-May 2021 (extended data SF2).”

# Peer review: Reviewer response for version 1

**DOI:** 10.21956/wellcomeopenres.18493.r44897

**Published:** 2021-07-26

**Authors:** Nei-Yuan Hsiao

**Affiliations:** 1 Division of Medical Virology, Department of Pathology, University of Cape Town, Cape Town, South Africa; 2 National Health Laboratory Service, Johannesburg, South Africa

Thank you for the opportunity to review this manuscript.

 In this study, Nimhans and colleagues performed a whole genome sequencing on virus identified among returning travellers, local surveillance effort and one outbreak in college students. The main purpose of the study is to describe the circulation of variants in Karnataka state and identify key mutations that may have clinical and public health importance.

 I think the work is of high quality and the manuscript both clearly presented the research conducted and correctly cites the international literature around this issue. As the study is descriptive in nature, I found the study design to be appropriate and the methodology widely adopted in the field so readily reproducible.

 The key interesting finding of the study is the identification of the B.1.36 lineage which I admit have not come across before. This lineage was both present in high enough proportion and contain significant mutations in spike to warrant attention and investigation. The authors did well to describe the extent of spread of this variant at the time, given the difficulty of the constantly changing nature SARS-CoV-2 lineage circulation.

 There are only a few minor suggested revisions on my part:

1. The local epidemic situation at the time of study could provide important context for which the genomic data can be interpreted. A simple epidemic curve or a description of number of people tested and positive in the region would assist readers further gauge the significance of the findings around novel variants.
2. The trees and phylogenetic analyses were solely based on local sequences. Even though for description purposes this is sufficient, adding related national and international sequences, especially those from potential sources of introduction, could vastly improve the interpretation.
3. Table 3 and 4 are superfluous and can probably just be described in text.
4. I would like to see the author discuss how this work shape their future priorities around genomic surveillance. The discussion suggested that the returning travellers are more readily sequenced than routinely diagnosed local cases. Would sequencing returning travellers from an important part of genomic surveillance? I think it would be good to add more voices in this discussion.

Is the work clearly and accurately presented and does it cite the current literature?

Yes

If applicable, is the statistical analysis and its interpretation appropriate?

I cannot comment. A qualified statistician is required.

Are all the source data underlying the results available to ensure full reproducibility?

Yes

Is the study design appropriate and is the work technically sound?

Yes

Are the conclusions drawn adequately supported by the results?

Yes

Are sufficient details of methods and analysis provided to allow replication by others?

Yes

Reviewer Expertise:

Clinical virology, Epidemiology, molecular diagnostics

I confirm that I have read this submission and believe that I have an appropriate level of expertise to confirm that it is of an acceptable scientific standard.

# Response

**Published:** 2022-01-30

**Authors:** Chitra Pattabiraman

We thank the reviewer for accepting and taking the time to review this manuscript during the pandemic and for their considered comments. We have made changes to the manuscript based on their recommendations. Please find a point-wise response to the comments below with changes to the manuscript indicated

**1. The local epidemic situation at the time of study could provide important context for which the genomic data can be interpreted. A simple epidemic curve or a description of the number of people tested and positive in the region would assist readers further gauge the significance of the findings around novel variants**

 We thank the reviewer for this suggestion. We have modified Figure1 to include an epi curve showing the daily confirmed cases in Bengaluru Urban district in the study period (Figure 1A). The average number of cases was 510, with a median test positivity of 1.2 %. The methods section now includes a data source for this graph. The following line has been added to the results section.

 “In the study period an average of 510 SARS-CoV-2 cases were detected daily in the district of Bengaluru Urban in the South Indian state of Karnataka (Figure 1).”

**2.The trees and phylogenetic analyses were solely based on local sequences. Even though for description purposes this is sufficient, adding related national and international sequences, especially those from potential sources of introduction, could vastly improve the interpretation.**

 We have performed a phylogenetic analysis using limited sequences of the B.1.36 lineage from all over India and across the world in this time period. This is included as part of extended data SF1. The following changes have been made-

 “Phylogenetic analysis of a subset of B.1.36 genomes (n=183) from across India and across the world showed that sequences from the study (n=70) both from circulating viruses as well as travel associated cases largely clustered together (extended data SF1 ^40^).”

 “The clustering of B.1.36 lineage from travellers with locally circulating viruses suggests either a common source of infection or infection with circulating lineages post-arrival (extended data SF1).

 3. **Table 3 and 4 are superfluous and can probably just be described in text.**

 Table 2 and 3 have been described in the text and moved to extended data table S6. Table 4 has been removed, and a more detailed summary count of Lineage B.1.36 from across the world is provided in extended data S8.

**4. I would like to see the author discuss how this work shape their future priorities around genomic surveillance. The discussion suggested that the returning travellers are more readily sequenced than routinely diagnosed local cases. Would sequencing returning travellers from an important part of genomic surveillance? I think it would be good to add more voices in this discussion.**

 The resources for sequencing are not available equally in all parts of the world and given the resource limitations amplified by the pandemic, it has been hard to argue for an investment in genomic surveillance when it is not always clear what to do with the data or who truly benefits from it. Nevertheless, we believe this is important and of local and global relevance.

 We have modified the last paragraph of the discussion as follows-

 “We believe that in regions where sequencing capacity is limited and sustained and continuous sequencing of local cases is a challenge, rapid and focussed sequencing of travellers and local outbreaks may serve as early warning systems for novel variants. Even as this conjecture remains to be tested, it is clear that rapid identification of such variants can aid in preparing the healthcare system for a surge in cases, suggest revisions to vaccines and diagnostic tests, inform the international community, and guide public health measures.”
